# Complete Genome Sequence of Clover Yellow Mosaic Virus Isolated from White Clover in Japan

**DOI:** 10.1128/mra.00324-22

**Published:** 2022-05-24

**Authors:** Masato Suzuki, Nozomu Iwabuchi, Yuji Fujimoto, Takumi Suzuki, Oki Matsumoto, Tomohiro Neil Motohashi, Akio Miyazaki, Kensaku Maejima, Shigetou Namba, Yasuyuki Yamaji

**Affiliations:** a Department of Agricultural and Environmental Biology, Graduate School of Agricultural and Life Sciences, The University of Tokyo, Tokyo, Japan; Queens College CUNY

## Abstract

Clover yellow mosaic virus (ClYMV) infecting white clover was isolated in Japan, and the complete genome sequence was determined.

## ANNOUNCEMENT

*Clover yellow mosaic virus* (ClYMV) is a member of the genus *Potexvirus* in the family *Alphaflexiviridae* (1). The genome is a positive-sense single-stranded RNA (2). ClYMV is an important pathogen of clovers; it causes yellow or light-green stripes and reduces clover winter hardiness and yield (3). ClYMV infects clover, broad bean, pea, alfalfa, Chenopodium album, chickweed, apple (3), *Verbena* spp. (4, 5), and tulips (6). ClYMV has been reported in North America (2, 3), Europe (4, 5, 7), and Oceania (8); however, its complete genome sequence has been reported for only two isolates from Canada (2) and Poland (5). The present study reports a complete genome sequence of ClYMV isolated in Japan.

**FIG 1:**
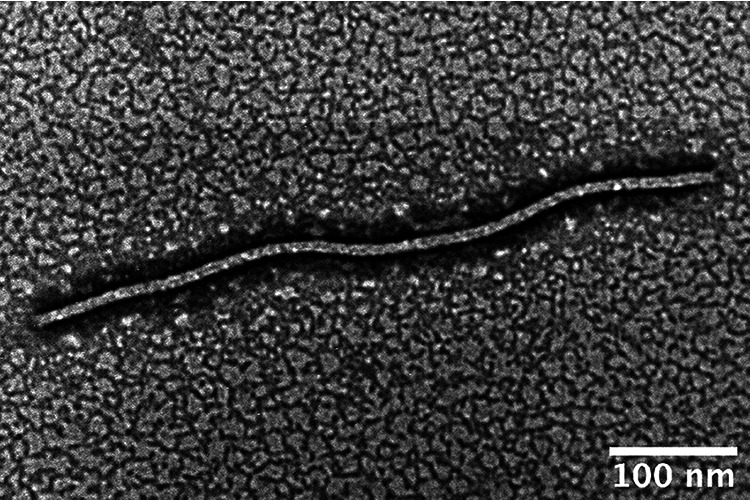
Electron micrograph of a flexuous filamentous potexvirus-like particle observed in crude sap from a symptomatic white clover leaf.

In 2021, white clover plants (Trifolium repens) with yellow mosaic symptoms were collected in Midori-cho (Nishitokyo, Tokyo, Japan). Crude sap from the symptomatic leaf was stained with 2% phosphotungstic acid. Transmission electron microscopy showed flexuous filamentous potexvirus-like particles (Fig. 1). Total RNA was extracted from the symptomatic leaf using a plant total RNA mini kit (Favorgen, Taiwan), and the DNA was eliminated using DNase I (Nippon Gene, Japan). Reverse transcription PCR (RT-PCR) was performed with primers specific to an internal region of the potexvirus replicase gene (9) (Table 1), as described previously (10). The amplified fragment was directly sequenced by Sanger sequencing using the same primers. A BLASTn search revealed that the sequenced 708 nucleotides (nt) shared 82.0% identity with partial sequences of ClYMV isolates.

**TABLE 1:** List of primers used in this study

Primer	Sequence (5′ to 3′)	Purpose	Reference
Potex 1	CAYCARCARGCNAARGAYSA	Amplification of an internal region of potexvirus replicase	Gibbs et al. (9)
Potex 2	TCDGTRTTDGCRTCRAADGT	Amplification of an internal region of potexvirus replicase	Gibbs et al. (9)
ClYMV RACE^*a*^ R1	CCTAAATCTTCCAGCAGGTC	5′ RACE	This study
ClYMV RACE R2	TACATTCTCATATTGGTCGC	5′ RACE	This study
GeneRacer 5′ primer	CGACTGGAGCACGAGGACACTGA	5′ RACE	GeneRacer kit (Invitrogen)
GeneRacer oligo(dT) primer	GCTGTCAACGATACGCTACGTAACGGCATGACAGTG(T)_18_	cDNA synthesis	GeneRacer kit (Invitrogen)
ClYMV 1F	GAAAACGAAACAAACCAAAACGAAAC	Amplification of the ClYMV genome	This study
KpGR3nest	GGGGTACCGCTACGTAACGGCATGACAGTG	Amplification of the ClYMV genome	Yusa et al. (12)
KpGR3nesF	CCGTTACGTAGCGGTACCCCTCAAACATTTGGCAATAAA	Cloning of the ClYMV genome into the pPPVOu vector	Yusa et al. (12)
ClYMV35S R	TTTGGTTTGTTTCGTTTTCCCTCTCCAAATGAAATGAAC	Cloning of the ClYMV genome into the pPPVOu vector	This study
35Spro F	TGGATTGATGTGACATCTCC	Sequencing by primer walking	This study
ClYMV 783F	GATTGACTGGCTGAGATTTG	Sequencing by primer walking	This study
ClYMV 1675F	CAATCCAAACCAACAAGTGC	Sequencing by primer walking	This study
ClYMV 3278R	GTGAGGTGATTGATCATAGC	Sequencing by primer walking	This study
ClYMV 3773F	TCCCTGTTGAGAATGAGAAC	Sequencing by primer walking	This study
ClYMV 4010R	TTCAGCCTGAACTCCTCAAG	Sequencing by primer walking	This study
ClYMV 4535F	CAGAAGCAATCATTCAAGGC	Sequencing by primer walking	This study
ClYMV 5279F	ACCTCCATACTACCTTACAC	Sequencing by primer walking	This study
ClYMV 6107F	TCAATGGACACTCAGCCTTC	Sequencing by primer walking	This study
ClYMV 6546F	TTTGGAACTATGCTCTCAGG	Sequencing by primer walking	This study

a RACE, rapid amplification of cDNA ends.

Next, we determined the complete genome sequence of the virus. Three cycles of single local lesion transfers were performed on Chenopodium quinoa leaves to obtain a ClYMV isolate (ClYMV-JPN-2021). Virion purification and phenol-chloroform RNA extraction were conducted as described previously (11). Prior to whole-genome amplification, the 5′-terminal sequence was determined. Using two reverse primers designed on 5′-proximal regions (Table 1), 5′ RACE and sequencing of the 5′ RACE product were performed as described previously (10). To obtain full-length cDNA from ClYMV-JPN-2021, reverse transcription was conducted using GeneRacer oligo(dT) primer (Invitrogen, USA), which hybridizes the 3′ poly(A) tail of the potexvirus genome. PCR was performed on the cDNA using a ClYMV 1F primer designed on the 5′-end sequence determined by 5′ RACE and a KpGR3nest primer designed on the GeneRacer oligo(dT) primer (12). The amplified ClYMV-JPN-2021 genome was inserted into the pPPVOu binary vector (13) as described previously (12), and six clones were sequenced by primer walking using the primers listed in Table 1. Using ATGC v4.3.5 software (Genetyx, Japan), all sequence reads from the six clones were trimmed and assembled into a single contig with 100% identity in each of the overlapping regions.

The complete genome sequence of ClYMV-JPN-2021 was 6,985 nt long with 47.8% GC content, excluding the 3′ poly(A) tail. The NCBI open reading frame (ORF) finder (https://www.ncbi.nlm.nih.gov/orffinder/) was used to predict five ORFs typical of potexviruses. Sequence identities between ClYMV-JPN-2021 and the other two ClYMV isolates (GenBank accession numbers D29630.1 and MT176428.1) were calculated using the MUSCLE algorithm (14) in the program SDT v1.2 (15). The analysis revealed nucleotide and amino acid identities of 77.5 to 78.6% and 85.2 to 85.4% for the replicase and 79.5 to 81.4% and 92.9 to 95.8% for the coat protein, respectively. According to the current sequence-based species demarcation criterion for the genus *Potexvirus* (16), ClYMV-JPN-2021 was identified as an isolate of ClYMV that is distantly related to the previously reported isolates.

### Data availability.

The ClYMV-JPN-2021 genome sequence has been deposited in the DNA Data Bank of Japan under the accession number LC682768.1.
